# Affective Destabilisation Associated With Tirzepatide in a Patient With Narcissistic Personality Traits

**DOI:** 10.1192/bjo.2026.11887

**Published:** 2026-06-30

**Authors:** Gayathri Rangith

**Affiliations:** Cygnet Health Care, Oldbury, United Kingdom

## Abstract

**Aims:**

Weight-loss medications such as tirzepatide are increasingly prescribed, yet their potential psychiatric effects remain under-recognised. This case explores diagnostic uncertainty between personality pathology and hypomania following significant weight loss and exposure to tirzepatide in an individual with pre-existing narcissistic personality traits. The aim was to examine whether behavioural escalation represented an exacerbation of personality traits alone or a superimposed affective episode temporally associated with medication use.

**Methods:**

A man in his late 40s with established Narcissistic Personality traits and no prior history of mood disorder was assessed following escalating behavioural concerns in the workplace. Prior to medication exposure, occupational functioning was stable, though interpersonal behaviour was characterised by inconsistency, criticality, and sensitivity to perceived errors in others.

Tirzepatide was initiated for weight loss at a starting dose of 2.5 mg and gradually escalated to 10 mg. Over approximately six months, the patient experienced rapid weight loss of 20 kg. Over the last three months of the treatment, marked behavioural changes were observed, including reduced need for sleep, increased energy, heightened grandiosity beyond baseline, impulsive decision-making, disinhibition, and poor insight into the impact of his actions on colleagues.

He demonstrated abrupt rule-breaking behaviour, including suspending colleagues without completing appropriate investigations or following established procedures, causing significant distress within the workplace. Symptoms were escalating rather than static. There were no psychotic symptoms, substance misuse, or alternative medical explanations identified.

**Results:**

The primary diagnostic considerations included exacerbation of narcissistic personality traits, hypomania, and medication-associated affective destabilisation. Several features supported hypomania over personality traits alone, including a clear change from baseline functioning, reduced sleep, increased goal-directed activity, behavioural disinhibition, and a marked reduction in insight. The temporal association with tirzepatide initiation and rapid weight loss raised the possibility of medication-related affective change in a psychologically vulnerable individual, despite no prior bipolar diagnosis.

**Conclusion:**

This case highlights the diagnostic complexity of distinguishing personality pathology from emergent mood disorder in the context of metabolic pharmacotherapy. Clinicians should remain vigilant for affective destabilisation in patients prescribed GLP-1–based weight-loss agents, particularly those with underlying personality vulnerabilities. Careful longitudinal assessment is essential before attributing behavioural change solely to personality traits.

